# Notch signaling in pediatric soft tissue sarcomas

**DOI:** 10.1186/1741-7015-10-141

**Published:** 2012-11-16

**Authors:** Rossella Rota, Roberta Ciarapica, Lucio Miele, Franco Locatelli

**Affiliations:** 1 Department of Oncohematology, Ospedale Pediatrico Bambino Gesù, IRCCS, Piazza Sant'Onofrio 4, Roma, 00165, Italy; 2 Cancer Institute, University of Mississippi Medical Center, 2500 N. State Street, Guyton 2 Building, Suite G751-05, Jackson, Mississippi, 39216, USA; 3 Dipartimento di Scienze Pediatriche, Policlinico San Matteo, IRCCS, Università di Pavia, Viale Camillo Golgi 19, Pavia, 27100, Italy

**Keywords:** soft tissue sarcoma, Notch, mesenchymal cells, γ-secretase, Synovial sarcoma, Ewing sarcoma, Rhabdomyosarcoma

## Abstract

Pediatric soft tissue sarcomas are rare tumors of childhood, frequently characterized by specific chromosome translocations. Despite improvements in treatment, their clinical management is often challenging due to the low responsiveness of metastatic forms and aggressive variants to conventional therapeutic approaches, which leads to poor overall survival. It is widely thought that soft tissue sarcomas derive from mesenchymal progenitor cells that, during embryonic life, have developed chromosomal aberrations with de-regulation of the main pathways governing tissue morphogenesis. The Notch signaling pathway is one of the most important molecular networks involved in differentiation processes. Emerging evidence highlights the role of Notch signaling de-regulation in the biology of these pediatric sarcomas. In this review, we present an outline of recently gathered evidence on the role of Notch signaling in soft tissue sarcomas, highlighting its importance in tumor cell biology. The potential challenges and opportunities of targeting Notch signaling in the treatment of pediatric soft tissue sarcomas are also discussed.

## Review

### Differences between adult and pediatric tumors

What makes the majority of pediatric tumors different from adult ones is their 'embryonal' origin. An inflammatory microenvironment and/or the age-dependent accumulation of genetic mutations and epigenetic alterations have a well-recognized importance in the pathogenesis of adult tumors. Conversely, in tumors arising in newborns and children, de-regulation of developmental pathways during embryonic life seems to play a major role. This view is also supported by the observation that the most aggressive pediatric tumors frequently harbor chromosomal translocations involving genes that regulate embryogenesis and tissue determination.

Several critical developmental pathways have been involved in pediatric tumor biology such as Hedgehog (Hh), Wnt and, more recently, Notch signaling. All are crucial regulators of differentiation, balancing proliferation versus differentiation and defining the tissue lineage commitment of precursor cells. Mutations of molecular components of Notch signaling have been involved in different genetic disorders [1-3]. As of this writing, Notch signaling de-regulation is recognized as a feature of several types of adult cancers [4-9]. However, the first evidence that human Notch1 is a proto-oncogene came from a predominantly pediatric malignancy, acute T-cell leukemia (T-ALL) [10]. Notch1 was subsequently shown to be the most commonly mutated oncogene in T-ALL [11]. Over the past few years, growing evidence also points to a role of abnormalities of Notch signaling in pediatric solid tumors. Recently, Notch signaling has been investigated, by our and other groups, in tumors of childhood that are thought to originate from mesenchymal progenitors, that is, soft tissue sarcomas (STS). The unresponsiveness to current conventional therapies observed for metastatic STS and the higher relapse rate seen for the translocation-bearing aggressive histological subtypes, together with the significant adverse effects of current therapy in young people, prompted the research community to seek new markers/targets for treatment of these tumors. In this context, therapies aimed at modulating the Notch signaling pathway are considered promising for tailored approaches. Notch inhibitors are currently being evaluated in a growing number of clinical trials, mainly in adults.

The present review aims at summarizing recent insights on the role of Notch signaling in pediatric STSs, highlighting the context/tumor-dependent role of specific Notch components. Current therapeutic strategies aimed at inhibiting Notch signaling and their potential pros and cons are discussed.

### Soft tissue sarcomas: a developmental defect?

**Figure 1:**
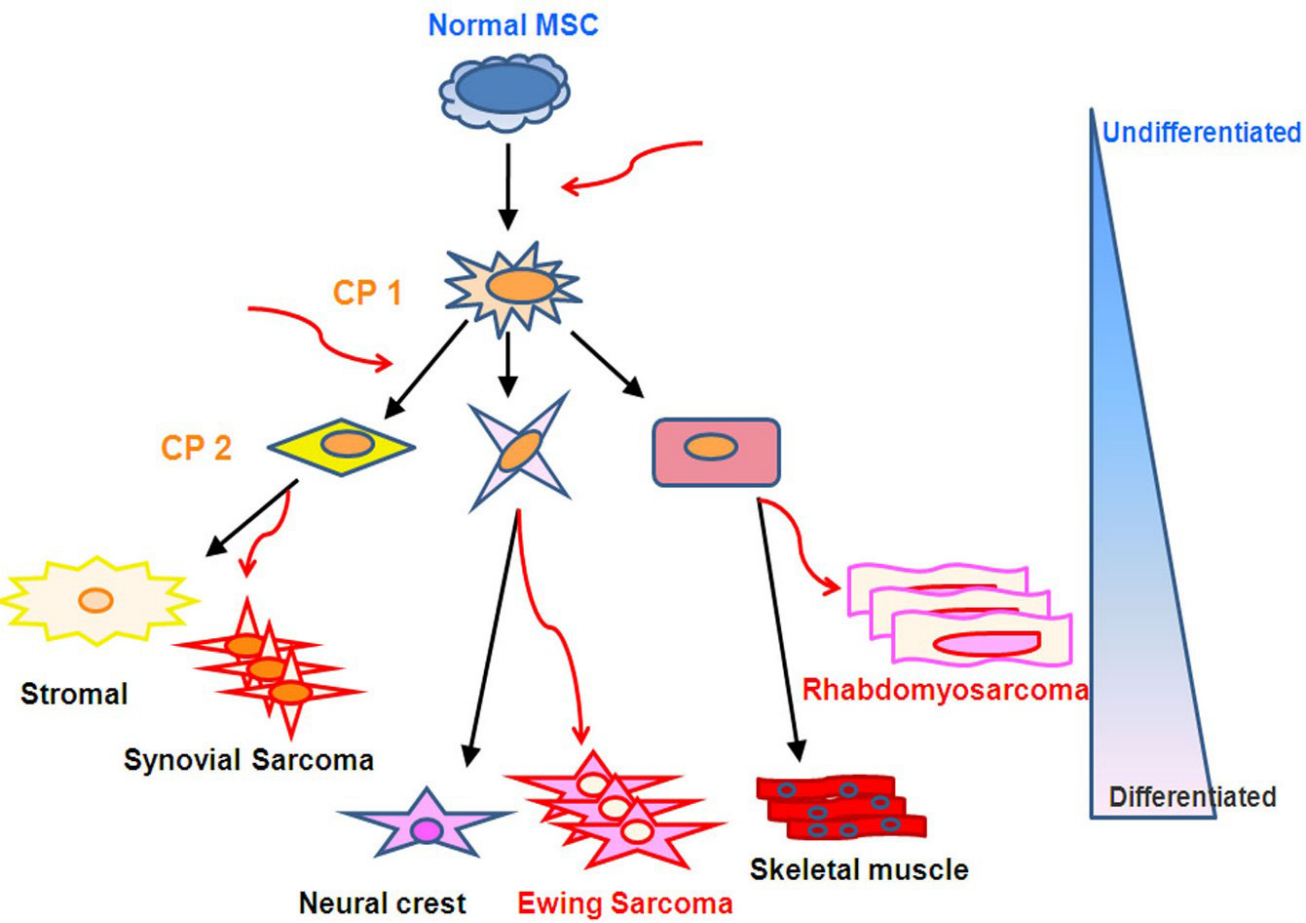
**Mesenchymal tissues differentiation and pediatric sarcomagenesis**. Schematic representation depicting how pediatric soft tissue sarcomas (STS) may be formed from a mesenchymal stem cell (MSC) through mutation and/or chromosomal translocation hits (red arrows). In normal developmental conditions, embryonic MSC undergo sequential steps of maturation towards a committed primary progenitor (CP 1) that may express markers of more than one tissue type. Terminal cell differentiation through more committed progenitors, reported as CP 2 in the figure, is obtained by sequential steps leading to the differentiated tissue formation. In the Figure are reported MSC-derived normal tissues such as stromal, neural crest and skeletal muscle tissues and the corresponding potential pediatric STS: Synovial Sarcoma, Ewing Sarcoma and Rhabdomyosarcoma. The stage of MSC maturation in which mutation/translocation occurs is indicative of tumor-tissue differentiation degree

Pediatric STSs consist of a group of heterogeneous malignancies of mesenchymal origin that accounts for 1% of all human cancers and up to 15% of all pediatric tumors (Figure 1). STSs represent a clinical challenge because, due to their infiltrating potential, they are generally difficult to eradicate surgically and, especially when metastatic at diagnosis, are unresponsive to conventional therapy [12,13].

Our growing knowledge of the molecular pathogenesis of STS suggests new antitumor treatments based on targeted molecular strategies. Consistent with the hypothesis that aberrant embryonic developmental molecular pathways may be involved in the development of pediatric STS, there is evidence that clonal cell populations harboring only one specific chromosomal translocation are maintained throughout tumor progression [14]. Is it possible that mesenchymal progenitor cells (MSC) that undergo a physiological cellular maturation become neoplastic at discrete stages of differentiation? Evidence for such a mechanism has been reported for Ewing sarcoma (ES) [15,16] and rhabdomyosarcoma (RMS) [17]. A high histological differentiation degree in STSs correlates with a good prognosis. It is conceivable that what determines the clinical failure of STS treatment is the presence of small populations of highly undifferentiated cells that have tumor-initiating potential and self-renewal capacity, and that are largely unresponsive to chemotherapy [17,18]. In this context, blocking developmental pathways involved in the maintenance of the stem cell compartment, such as the Notch pathway, may be an attractive strategy to improve the clinical management of STS.

Indeed, recently we and other groups have shown pre-clinical evidence that Notch signaling modulation has anti-tumor effects in the most common pediatric STS such as ES and RMS.

### Notch signaling pathway

The Notch signaling is a close range cell-to-cell communication system that allows the crosstalk between different contiguous cellular compartments in the embryo and in post-natal life. Notch signals regulate the determination/maintenance of cellular identity. In mammals, the Notch signaling pathway consists of four trans-membrane receptors, Notch1 to Notch4, encoded by homologous but different genes (reviewed in [4,19,20]). Notch receptors include an extracellular region, a trans-membrane region, and an intracellular domain (ICD) [20]. The Notch receptors are activated upon binding to trans-membrane ligands of the Delta-Serrate/Jagged families. Humans and rodents express at least three Delta-like (DLL) ligands, 1, 3 and 4 and two Serrate/Jagged family ligands, JAG1 and 2. Notch ligands are expressed on the surface of cells contiguous to receptor-expressing cells. Ligand binding to a Notch receptor results in two sequential receptor cleavages operated by specific proteases, an ADAM protease at the cell surface and γ-secretase in the transmembrane domain. Cleavage by γ-secretase (GSI) releases Notch ICD into the cytoplasm. The Notch ICD is the 'active' fragment that migrates to the nucleus. In the nucleus, Notch ICD replaces the co-repressor complex on a DNA-binding transcription factor, CSL/RBP-Jk (CBF1/RBPjκ/Su(H)/Lag-1). The Notch-CSL complex triggers the transcription of Notch target genes. These include, among others, the basic helix-loop-helix (bHLH) transcription factors of the *Hes *and *Hey *(HESR) families working as transcriptional repressors [21]. The Notch ICD once released into the cytoplasm can be post-translationally modified and/or can interact with several molecules that either amplify or dampen Notch signaling [19]. These include, for example, prolyl isomerase Pin1 [22] and Nemo-like kinase NLK [23]. In particular, the C-terminal proline, glutamic acid, serine and threonine degradation (PEST) domain is a target for ubiquitylation favoring ICD proteolytic degradation (reviewed in [4,19,20]). The complexity of the Notch system is further enhanced by non-canonical signaling. This includes cis-inhibition by Notch ligands expressed on the same cell as the receptors, Notch activation by non-canonical ligands, ligand-independent Notch activation and CSL-independent signaling [24]. Cytoplasmic Notch signaling mediated by the mTOR complex has been demonstrated in some cells, such as T regulatory cells [25]. The choice among these different manners of activation and the expression of specific Notch receptor paralogs and/or ligands during a specific cellular program is context-, cell- and time-dependent during both embryogenesis and post-natal life. Moreover, Notch paralogs expressed in the cell can behave in opposite manners [19]. Further levels of regulation are obtained before cell membrane localization of Notch receptor molecules through the glycosylation of the Notch extracellular domain in the Golgi complex by enzymes such as POFUT-1 (O-fucosyltransferase) and Manic, Lunatic and Radical Fringe (MFNG, LFNG and RFNG, respectively, O-fucosyl, β-1-3 N-acetylglucosaminyltransferases), which affects its affinity and binding to specific ligands. For instance, LFNG-mediated modifications in most cells reduce the affinity for Jagged family ligands but not Delta family ligands. Although this complexity may appear daunting, it offers multiple opportunities to modulate signal intensity at different levels or through different Notch receptors, thus acting on different aspects of tumor progression. One of the most studied inhibitory approaches is the use of GSI, first investigated in Alzheimer's disease, that prevent the cleavage of Notch receptors and subsequent Notch signaling activation. GSIs are being, or have been, investigated in several anti-cancer clinical trials.

Contrary to what is observed in T-ALL that often is triggered by a Notch1 receptor mutation, in solid cancers, few genetic alterations have been found so far in Notch signaling components. In many solid tumors, Notch signaling de-regulation may be a consequence of the primary oncogenic mutation(s) rather than the main causative event. However, in many such malignancies Notch signaling seems to play a general role in the maintenance of tumor phenotype and especially in the survival and self-replication of tumor-initiating cells. Additionally, non-cell autonomous roles of Notch signaling in tumor stroma, in endothelial cells and in the immune system can also contribute to tumor survival and recurrence (reviewed in [26]). In some situations, individual Notch paralogs have been found to have tumor-suppressive properties. The best-documented example is the role of Notch1 in squamous epithelia [27]. In some cases, these effects as well as non-cell autonomous result from disruption of epithelial barrier functions in the absence of Notch, which in turn promotes chronic dermal inflammation that predisposes to tumorigenesis [28].

### Notch signaling in synovial sarcoma

Synovial sarcoma (SS) develops in adolescents and young adults and has an aggressive behavior with high metastatic potential [29]. It accounts for 7% to 10% of all STS [30]. SS was initially termed 'synovial' because it is found in the soft tissue adjacent to joints of young adult patients and resembles developing synovial tissue. However, SS is frequently observed in extra-synovial locations such as kidney, lung and heart and recent findings suggest its derivation from MSCs [30,31]. The majority of SSs harbors the chromosomal translocation t(X;18) (p11;q11) between the Synovial Sarcoma translocation, chromosome 18 gene (*SS18*, previously *SYT*) on chromosome 18q11 and either Synovial Sarcoma, × breakpoint 1, 2 or 4 (*SSX1, SSX2*, or *SSX4*, respectively) genes on chromosome Xp11. The SS18-SSX fusion onco-proteins contain the transcriptional activation domain of SS18 fused to the repressor domain of SSX but lack a DNA-binding domain [32]. They form non-physiologic protein complexes that are absent in normal cells. The identification of their target genes is difficult because of the lack of direct binding to DNA. Expression of SS18-SSX onco-proteins is necessary and sufficient to cause malignant transformation. Indeed, rat fibroblasts expressing exogenous SS18-SSX showed a transformed behavior [32]. Conditional overexpression of SS18-SSX2 in transgenic mice results in SS formation but only when the transgene is expressed in mesenchymal-derived myogenic progenitors [29]. Finally, knockdown of SS18-SSX in SS cell lines induced loss of differentiation abilities into mesenchymal-derived tissue, strongly supporting the hypothesis of a MSC origin for this tumor [30].

To date, a direct mechanistic link between SS biology and Notch signaling has not been clearly demonstrated; however, some findings suggest that Notch signaling could be involved in SS (Table 1). In a pioneering study by Francis and colleagues, gene expression analysis was carried out in a large set of STS, among which were 31 SS primary samples [33]. In an effort to identify diagnostic marker genes that could discriminate among the different subtypes of STS, a very highly heterogeneous group of tumors, 4,000 genes were found to be differentially expressed in SS. These included the major developmental pathways such as Hh, Wnt, tumor growth factor β (TGFβ), chromatin remodeling complexes and Notch. In particular, *Notch1, JAG1 *and the *transducin-like enhancer (TLE) of split *genes were up-regulated in SS. *TLE *genes are known Notch targets, and encode a family of four master transcriptional regulators (TLE1 to 4), highly conserved among species. *TLE *genes are expressed during embryogenesis and work in concert with the Wnt/β catenin pathway, known to be pro-oncogenic in SS [34,35]. In particular, the over-expression of TLE1 appears to be a reliable marker to discriminate SS from other types of STS, independently from the type of SSX fusion and the degree of tumor differentiation [36-38], although a study showed TLE1 to be expressed also in other non-synovial STS [39]. TLE1, an evolutionarily conserved Notch effector, mediates the anti-differentiation functions of Notch signaling in neuronal cells. There, it works as an important co-repressor by facilitating the binding of HES proteins (direct Notch transcriptional targets) to promoters of target genes to allow gene repression [40]. Therefore, the over-expression of TLE1 in SS may mediate similar effects of Notch in SS and deserves further investigation.

**Table 1:** Notch signaling in STS.

Tumor	Results	Notch componentinvolved	Role of Notch signaling	Reference
Synovial sarcoma		Notch1, JAG1 and TLEs over-expression	Pro-tumor	[33]
		TLE1 over-expression	Pro-tumor	[36-39]
	Prevents EGR1 expression Reduces apoptosis Promotes cell growth Favors epigenetic gene repression	TLE1 over-expression	Pro-tumor	[41]
				
Ewing's sarcoma	Prevents differentiation Increases cell proliferation Decreases apoptosis Supports tumor growth *in vivo*	Manic Fringe expression Notch1 ICD over-expression	Onco-suppressor Regulates differentiation	[51]
	In p53 wild-type tumor cells: Induces p53 Blocks cell proliferation Inhibits soft agar colony formation	JAG1 and HEY1 down-regulated by EWS-FLI1 and Notch3	Onco-suppressor	[52]
	Prevents cell proliferation	Notch1 and Notch3	Onco-suppressor	[54]
				
Rhabdomyosarcoma	Supports cell proliferation Inhibits apoptosis Prevents differentiation	HES1 over-expression in RMS primary samples and in cell lines	Pro-tumor	[68]
	Increases cell migration Increases cell invasion	Notch2 and HEY1 over-expressed in patients with alveolar RMS and embryonal RMS	Pro-tumor	[69]
	Increases cell proliferation Supports tumor growth *in vivo*	Notch1 ICD and HEY1 over-expressed in primary embryonal RMS and cell lines	Pro-tumor	[70]
	Prevents differentiation Supports cell proliferation Supports tumor growth *in vivo*	Notch3 ICD and HES1 over-expressed in alveolar RMS and embryonal RMS cell lines	Pro-tumor	[71]
	Supports cell proliferation and anchorage-independence *in vitro *and tumor growth *in vivo*	RBP-Jκ over-expressed in embryonal RMS primary tissues and cell lines	Pro-tumor	[76]

Very recently, a functional role for TLE1 in SS pathogenesis has emerged from the observation that SSX18-SSX proteins behave as a scaffold to bridge TLE1 and the DNA-binding protein activating transcription factor 2 (ATF2) [41]. The physiologic role of ATF2 as a master transcriptional activator is reversed in the non-physiologic, pro-oncogenic complex SS18-SSX/TLE1/ATF2. The latter recruits histone deacetylases (HDACs), and retaines the co-repressor ability of TLE1 to repress the transcription of ATF2-induced genes, such as cell cycle regulator and apoptotic genes, resulting in tumor cell survival. Conversely, disruption of the SS18-SSX/TLE1/ATF2 complex by silencing of SS18-SSX led to cell cycle arrest and cell death. One of the targets repressed by the oncogenic complex SSX/TLE1/ATF2 is the *Early Growth Response-1 (EGR1*) gene, a tumor suppressor gene that regulates cell growth and differentiation. *EGR1 *was previously demonstrated by the same authors to be maintained at low levels in SS by the interaction of SS18-SSX with Polycomb proteins from the Polycomb Repressor Complex 2 (PRC2), such as Enhancer of Zeste Homolog 2 (EZH2) and PRC1, such as BMI1 polycomb ring finger oncogene (BMI1) [42]. The knockdown of TLE1 increased the transcription of *EGR1 *and decreased the levels of histone H3 trimethylation on Lysine 27 (H3K27me3), which is a mark of EZH2 activity [41]. Taken together, these findings suggest that TLE1, a known effector of Notch signaling, plays a fundamental role in the SS18-SSX epigenetic regulation of gene expression in SS. Future investigations could elucidate whether TLE1 has the same function in normal developing cells.

Interestingly, when EGR1 was re-expressed by a gain-of-function approach in SS cells, it induced the transcription of *Phosphatase and tensin homolog deleted in chromosome 10 (PTEN*) gene, which, in turn, favored the pro-apoptotic effects of HDACs inhibitors [42]. *PTEN *has been reported as mutated in one out of four samples from SS patients [43]. A link between PTEN expression and Notch signaling has been demonstrated in different tumor contexts. *PTEN *has been shown to be down-regulated after Notch1 ICD over-expression in hypoxic mesothelioma cells, even though no evidence for a direct CSL/RBP-Jκ-dependent effect was reported [44]. In pancreatic cancer cells, Notch signaling seems to regulate the phosphorylation rather than the transcription of PTEN [45]. Moreover, it has been recently reported that in normal and cancerous thymocytes loss of PTEN expression is partly due to HES1-dependent repression, suggesting a Notch-mediated indirect regulation in this cellular context [46,47]. However, since Notch1 and JAG1 are up-regulated in SS [33], the possibility that the down-regulation of PTEN observed in these tumor may be, at least in part, related to Notch1 signaling de-regulation deserves further investigation.

### Notch signaling in Ewing Sarcoma

The ES family tumors include sarcomas affecting bone and soft tissues in children and adolescents. They presumably derive from a MSC precursor and some of them express neuroectodermal markers such as neuron-specific enolase [48]. More than 80% of ES express EWS-FLI1 chimeric onco-proteins generated by the fusion of the *ES breakpoint region (EWS*) gene on chromosome 22q12 with genes of the *E transformed specific transcription factor (ETS*) family, mostly the *Friend leukemia virus integration 1 (FLI1*) gene on chromosome 11q24. These proteins act as transcription factors with different transcriptional abilities and gene targets compared to wild-type single products. EWS-FLI1 proteins, exogenously expressed in murine fibroblasts or present in ES cell lines, stimulate the transcription of MFNG, the enzyme that regulates the glycosylation of Notch receptors and, therefore, their affinity for ligands [49,50]. Based on these results, the involvement of Notch signaling in ES pathogenesis was recently investigated (Table 1) [51]. Ten EWS-FLI1-expressing primary ES samples and two cell lines evaluated in this work showed the expression of at least one of the Notch receptors and several ligands, while all expressed the Notch target gene *HES1 *and the glycosylation enzyme MFNG. HES1 expression in ES cell lines was inhibited via expression of a dominant-negative Notch1 or the use of a GSI, while it was increased after expression of active Notch1 ICD. However, the two ES cell lines used in this work behave in a different manner after Notch inhibition, only one of them showing reduction of cell proliferation and cell apoptotic rate. Inhibition of Notch signaling did not result in reduced tumor growth *in vivo *but rather in neuroectodermal differentiation of tumor xenografts. Therefore, the authors suggested that Notch signaling activation is responsible for the loss of differentiation in ES but it does not play a direct pro-tumorigenic role [51].

More recently, Ban *et al*., investigating the role of EWS-FLI1 in p53 induction and cell cycle arrest in ES cells, discovered a link between the fusion onco-protein and Notch signaling [52]. Indeed, upon EWS-FLI1 silencing, wild-type p53 ES cells showed p53 activation and triggering of the molecular cascade involving the cyclin-dependent kinase (CDK) inhibitor p21^Cip1 ^leading to cell growth arrest followed by apoptosis. The gene expression profiling of EWS-FLI1-depleted cells, analyzed in order to investigate the molecular pathways involved in p53 induction, showed the induction of both the Notch ligand JAG1 and the Notch target gene *HEY1. HEY1 *silencing counteracted the induction of p53 in response to EWS-FLI1 depletion, while forced expression of *HEY1 *was sufficient to induce p53 nuclear accumulation also in EWS-FLI1-expressing cells resulting in cell cycle arrest. This observation strongly suggests that EWS-FLI1 down-regulation triggers a HEY1-dependent p53 induction, blocking cell proliferation. Apoptotic pathways activation appeared to be mediated by other mechanisms. Subsequent studies demonstrated that *HEY1 *transcription is due to activation of the Notch3 receptor, the most highly expressed Notch receptor in both ES primary tumors and cell lines, by JAG1 (Jagged1). Jagged1 over-expression was observed after EWS-FLI1 knockdown. Notch3 signaling was inhibited through either JAG1 silencing, GSI or over-expression of Notch negative regulator NUMB. Interestingly, even the role of NUMB in the degradation of Notch3 seems to be cell/context-dependent underscoring the complexity of Notch signaling in normal and pathological contexts [53]. All these approaches resulted in reduction of both p53 and p21^Cip1 ^up-regulation. Conversely, forced expression of either JAG1 or DLL1 was capable of inducing p53 expression and nuclear accumulation, and HEY1 silencing reversed this effect indicating that the induction of p53 is HEY1-dependent in this context. Finally, Ban and colleagues identify a potential EWS-FLI1 binding site on the *JAG1 *promoter that could repress the expression of *JAG1*, as suggested by luciferase experiments. Therefore, in ESs that retain wild-type p53, Notch signaling seems to act as a tumor suppressor rather than as an oncogene, as reported for the majority of adult epithelial tumors. This would explain the failure of tumor growth inhibition observed in the previous report [51]. Importantly, differently from ES cells depleted of EWS-FLI1, HEY1 was not modulated after Notch inhibition in ES cells expressing EWS-FLI1, suggesting that Notch signaling is inactive in the presence of the fusion onco-protein. In a subsequent manuscript, the same group studied the activation status of Notch signaling in ES primary tumors by immunohistochemistry in order to identify the cause of the high transcriptional expression of Notch target gene *HES1 *found in ES tumors [54]. They noticed that, although the mRNA of at least one Notch receptor was expressed and that of *HES1 *was up-regulated, Notch signaling appeared to be inactive in a set of 22 of 24 ES samples. Indeed, the Notch cleaved products, that is, Notch ICDs, and HES1 protein, were not present in the nucleus, as demonstrated by the absence of nuclear staining. Consistent with this observation, the high mRNA expression of HES1 found in all the samples was not correlated with Notch receptor mRNA expression. HES1 expression was independent of both EWS-FLI1 expression and Notch signaling inhibition achieved with different means, nor was it induced by expression of Notch1 or Notch3 ICD, suggesting that it is regulated by other pathways in ES cells. Moreover, while Notch1 ICD and Notch3 ICD over-expression was sufficient to prevent the proliferation of ES cells, blockade of HES1 did not have any effect.

A recently discovered link between ES pathogenesis and epigenetic pathways could have implications for Notch signaling inactivation in this tumor [55]. Lysine-specific demethylase 1 (LSD1 or KMD1A) was shown to be over-expressed in 59 ES and 7 rhabdomyosarcoma (RMS) primary samples compared to normal MSCs, as already reported for primary samples of RMS and SS [56]. Pharmacologic inhibition of LSD1 induced p53 expression and prevented proliferation in several ES cell lines, re-establishing the H3K4 methylation, as reported for SS cell lines [55]. In addition to having a primary role in epithelial-mesenchymal transition (EMT), LSD1 stimulates cell proliferation and survival by binding histone deacetylase complexes and specifically demethylating both Lysine 4 and Lysine 9 on histone H3 (H3K4 and H3K9). LSD1 works by forming co-repressor complexes with HDACs, such as sirtuin 1 (SIRT1), and inhibits the transcription of Notch target genes such as *HES1 *in human normal cells, and HES1 and HEY1 in murine normal cells [57]. This finding has been previously reported in murine embryos, where LSD1 represses the expression of HEY1 during brain development [58] and Enhancer of Split (a corresponding *HEY1 *gene) during *Drosophila *development [59]. The working model involves binding of LSD1/SIRT-1 complexes to CSL/RBP-Jκ during repression of HES1 and release of LSD1/SIRT1 complexes from CSL by Notch ICD. Therefore, the inhibition of LSD1 in ES could remove the brake for the transcription of Notch target gene *HEY1 *even in the presence of EWS-FLI1, as suggested by the expression of p53, thus mimicking and/or augmenting the effects of EWS-FLI1 silencing.

### Notch signaling in Rhabdomyosarcoma

RMS is the most common pediatric STS accounting almost for 7% to 8% of all childhood malignancies [60]. It includes two major histopathological pediatric subtypes, embryonal and alveolar, presenting different genetic abnormalities. Embryonal RMS occurs in about 70% of cases and has a good prognosis if it is non-metastatic at diagnosis. In contrast, alveolar RMS is often associated with a worse prognosis. In about 75% of cases, the alveolar form is characterized by specific chromosomal translocations such as t(2;13) or t(1;13) that produce paired box 3-*for*khead box O1 (PAX3-FOXO1) or PAX7-FOXO1 fusion onco-proteins. Their expression is correlated with poor prognosis, with an approximately 25% five-year overall survival rate for both PAX-FOXO1 positive alveolar and metastatic patients [61-64]. RMS is thought to derive from skeletal muscle progenitors that, although they retain expression of skeletal muscle markers such as MyoD and Myogenin, have lost the ability to differentiate and proliferate indefinitely. Therefore, strategies aimed at re-establishing differentiation programs are thought to have anti-cancer potential. Notch signaling is one of the major regulators of embryonic and post-natal skeletal muscle differentiation [65-67] and, therefore, could be implicated in RMS development. Consistent with this hypothesis, Sang *et al*. demonstrated that the Notch target gene *HES1 *was over-expressed in RMS primary tumors and cell lines compared to normal muscle, and its inhibition through a dominant-negative HES1 form promoted skeletal muscle-like differentiation of RMS cells (Table 1) [68]. Inhibition of Notch signaling with GSIs phenocopied this pro-myogenic effect and lowered HES1 expression, suggesting that HES1 de-regulation in RMS is Notch-dependent. In view of this important result, three reports published by our group and others in the last two years have confirmed a role of Notch signaling in RMS pathogenesis, unveiling several Notch-regulated mechanisms [69-71]. In 37 primary RMS samples, whether alveolar or embryonal, transcript levels of Notch2 and HEY1 were found significantly up-regulated, while Notch3 was slightly increased as compared to both adult and fetal muscle [69]. No significant over-expression was observed for either Notch1 or Notch4 mRNAs whereas a modest up-regulation of HES1 was found only in embryonal RMS samples. However, immunohistochemical nuclear staining for HES1 showed that it was over-expressed in the majority of tumor samples. HES1 levels correlated with tumor migration and invasive features of RMS cell lines *in vitro*, being modest in embryonal subtype cells with low invasive activity, high in highly invasive PAX7-FOXO1 alveolar cells and very high in PAX3-FOXO1 alveolar cells with the highest invasiveness. Treatment with GSIs or with a dominant-negative form of MAML1, a canonical Notch nuclear co-activator, prevented cell invasion with no effects on cell proliferation. More recently, HEY1 mRNA levels were shown to be significantly higher in the embryonal compared with the alveolar subtype in four RMS cell lines and a previously published cohort of primary RMS [62,70]. Consistent with a role of Notch1-target gene *HEY1 *in muscle progenitors [72,73], Notch1 protein levels were shown to be significantly up-regulated in embryonal RMS. A role of Notch1 and HEY1 over-expression in embryonal RMS is supported by the inhibition of cell proliferation in tumor cell lines depleted of either Notch1 or HEY1. Moreover, sustained silencing of HEY1 increased the expression of the myogenic differentiation factor Myogenin in embryonal RMS cells cultured in a medium that promotes cell proliferation (growth medium; GM), and even more in medium that promotes myotube fusion (differentiation medium; DM). However, no overt phenotypic signs of muscle-like differentiation and myotube fusion were detected after HEY1 knockdown, suggesting a role for the Notch1-HEY1 axis in the regulation of proliferative rather than differentiative pathways in embryonal RMS cells. Similar *in vitro *effects were observed after pharmacologic treatment with a GSI and were reversed by forced expression of an exogenous Notch1 ICD. Notch1 knockdown in RMS cell lines reduces or abolishes tumorigenicity in xenografts. This effect is confirmed with a GSI that decreased Notch1 ICD in tumor samples. Our investigations on Notch pathway in RMS biology, published some months later, expand the scenario, implicating Notch3 as a major cause of the inability of RMS cells to differentiate, irrespective of their subtype. In a set of three embryonal and two alveolar cell lines over-expressing transcripts of Notch1, Notch3 and HES1 compared to normal human skeletal myoblasts, the cleaved forms of these receptors, Notch1 ICD and Notch3 ICD were detected in the nucleus. Interestingly, tumor cells had similar Notch2 mRNA and protein levels to normal myoblasts, but the latter showed higher Notch2 ICD levels. Notch2 ICD was detected in nuclear extracts of all the cell lines studied, suggesting that this signal may be active in these RMS cell lines. Consistent with data from Sang *et al*., HES1 was up-regulated at the mRNA and protein levels in all the cell lines examined. Notch3 silencing in both one embryonal and one alveolar RMS cell line resulted in the formation of myotube-like structures expressing markers of terminal skeletal muscle differentiation such as myosin heavy chain and troponin. This finding is consistent with a role of Notch3 activation/expression in the myoblast-to-myofibroblast trans-differentiation induced by TGFβ treatment [74]. In agreement with these observations, Notch3 knockdown noticeably led to a decrease in HES1 expression associated with the activation of myogenic pathways necessary for terminal differentiation such as p38 and AKT. Moreover, JAG1 depletion strongly reduced both Notch3 ICD and HES1 levels, suggesting that Notch3 activation results, at least in part, from binding to Jagged-family ligands rather than from activating mutations. High throughput sequencing analysis on 75 ES and 89 RMS samples did not demonstrate any Notch1 mutations [75]. Notch1 silencing had a lesser effect on the differentiation of the embryonal cell line, slightly increasing the expression of myogenin, and no effect in the alveolar cell line. These results are in keeping with data from Linardic and colleagues, establishing that Notch1 signaling controls neither cell differentiation nor HES1 expression in embryonal RMS [70]. Strikingly, Notch2 knockdown reduced myogenin expression and promoted HES1 expression, indicating that Notch2 could play an opposite role in RMS cells compared to Notch3, as already suggested for skeletal muscle tissue commitment [74]. The triggering of differentiation upon Notch3 knockdown was associated with cell cycle arrest, p21^Cip1 ^induction and a decrease in the levels of kinases regulating cell proliferation such as ERK-1 and -2. All these effects were mimicked in wild-type cells or reinforced in Notch3-depleted cells by HES1 silencing, whereas HES1 over-expression partly reversed the effects of Notch3 knockdown. Consistent with the observations of Sang *et al. *[68], these results strongly indicate that the *in vitro *anti-differentiative and pro-tumorigenic role of Notch3 in RMS is, at least in part, due to the induction of HES1 expression. Finally, Notch3 depletion, even only in a fraction of cells, inhibited the growth of alveolar RMS tumors xenografted in immune-compromised mice. Very recently, Nagao and colleagues showed that the CSL/RBP-Jk is necessary for the growth of embryonal RMS cells *in vitro *and *in vivo *and that its function is Notch activation-dependent [76]. All these results support a major role for the Notch signaling pathway in the maintenance of the malignant phenotype in RMS.

### Notch signaling inhibition in cancer treatment: from preclinical studies to clinical evidence

Notch inhibition may represent a powerful approach to restrain tumorigenesis of STSs in which Notch signaling is over-active, such as RMS [69-71], and potentially, SS. Different classes of inhibitors have been tested in preclinical studies aimed at evaluating the feasibility of Notch inhibition as an anti-cancer strategy. These include GSIs [77], specific anti-Notch antibodies [78,79], Notch ligand decoys [80], and inhibitors of the Notch transcription complex [81]. Due to the role of Notch signaling in preventing differentiation and maintaining stem cell populations, these inhibitors are expected to induce differentiation and prevent proliferation and metastasis. Among preclinical Notch inhibitors, GSIs and some ligand mAbs have been tested in clinical trials for the treatment of human cancers.

GSIs, originally developed to block the cleavage of β-amyloid precursor protein in Alzheimer's disease, inhibit the final cleavage that produces the ICD of Notch receptors. However, they also inhibit the cleavage of many other γ-secretase-targeted proteins [82]. Thus, GSIs are not technically specific Notch inhibitors but their *in vivo *toxicity appears to result exclusively from Notch inhibition. Indeed, secretory diarrhea secondary to goblet cell metaplasia of the small intestine is due to inhibition of Notch1 and Notch2 cleavage in intestinal epithelial stem cells, and is mimicked by double knockout of Notch1 and Notch2 in these cells [83,84]. GSIs, at least theoretically, are not specific for individual Notch receptors. However, currently available investigational GSIs belong to several different chemical series, and the specificity of different classes of GSIs has not been studied in detail. Most biochemical studies on GSIs have traditionally focused on Notch1. The widespread assumption that all GSIs are pharmacologically equivalent and inhibit all Notch paralogs may be unwarranted. Harrison *et al. *[85] reported that Notch4 is resistant to two commercially available GSIs. Whether clinical GSIs also inhibit different Notch paralogs with different potencies remains to be determined. Initial results from clinical studies on patients with Alzheimer's disease reported target-mediated side effects of GSIs, such as secretory diarrhea, nausea and fatigue [86]. Nevertheless, a re-evaluation of case reports showed some therapeutic benefits and relatively low toxicity. For oncologic indications, intermittent administration of GSIs has been shown to decrease toxicity significantly. MK-0752 is an oral GSI. Its safety and efficacy as an anticancer drug have been tested in clinical trials with both adult and pediatric patients (Table 2) [87,88]. In a phase I study in children with recurrent central nervous system (CNS) malignancies, MK-0752 was well tolerated at the dose and schedule recommended for phase II study progression [87]. Two of nine patients experienced prolonged disease stabilization. Importantly, only in the two responders did MK-0752 decrease the levels of Notch1 ICD in post-treatment peripheral blood mononuclear cells [87]. This finding underscores a fundamental issue in the development-targeted agents, including Notch inhibitors. Robust biological markers demonstrating target inhibition should be included in all clinical trials of such agents for the results to be interpretable. Ideally, target inhibition in tumor tissue should be documented. In a phase I clinical trial conducted in adult patients with solid tumors, MK-0752 demonstrated good tolerability and evidence of Notch pathway inhibition using a once-per-week dosing schedule. Preliminary evidence of efficacy was observed mainly in patients with gliomas. Conversely, there was little evidence of efficacy of MK-0752 as a single agent in patients with extra-cranial cancers, this indicating the need for rational, mechanism-based combinations [88]. The importance of combination approaches is illustrated by a recently closed pilot pre-surgical clinical trial of MK-0752 in estrogen receptor (ER+) breast cancer ([89] and manuscript in preparation). In this study, which was based on extensive preclinical data documenting cross-talk of Notch with ER [90,91], MK0752 GSI was administered after two weeks of endocrine therapy with tamoxifen or letrozole, concomitantly with continued endocrine therapy. Under these conditions, no diarrhea was observed and Ki67 reduction compared to endocrine therapy alone was seen in 17 of 20 patients. Induction of apoptosis (as detected by upregulation of the pro-apoptotic mediator NOXA), was seen in 15 of 20 patients. Notch pathway inhibition by GSIs in tumor tissue was documented using multiple QRT-PCR assays on tumor biopsies taken at diagnosis, after endocrine therapy alone and after the addition of GSI. A chemically different GSI, RO4929097, has shown antitumor activity in animal models with a concomitant differentiated histologic profile, typical of Notch inhibition. Because of its promising preclinical activity and tolerability RO4929097 has been studied in phase I clinical trials [92]. Tolcher *et al. *have reported that in a phase I study RO4929097 was well tolerated in adult patients with refractory metastatic or advanced solid tumors and some evidence of antitumor activity was observed [93]. However, Strosberg *et al. *in a phase II study showed that patients with refractory metastatic colorectal cancer treated with RO4929097 did not have radiographic responses, suggesting that RO4929097 at the study dose and schedule has minimal single agent activity in this malignancy [94]. It should be noted that RO4929097 has auto-inducing properties (it induces its own liver metabolism) and this may represent a pharmacokinetic liability. Nevertheless, to expect single agent activity in early clinical trials with developmental pathway inhibitors may be unrealistic. Unlike cytotoxic agents, drugs targeting developmental pathways are not relatively non-specific poisons and do not often kill target cells at pharmacologically attainable doses. What many of these agents do is to reset cell fate programs increasing sensitivity to differentiation stimuli, growth arrest stimuli or cytotoxic stimuli. Additionally, it is likely that the main target of these agents is not bulk tumor cells but tumor-initiating cells. Therefore, the main therapeutic effect of these agents may lie in prevention of recurrence rather than rapid tumor shrinkage. This was demonstrated in a recent study in Her2/Neu positive xenografts, in which two chemically distinct GSIs (MRK003 and LY411,575) were studied alone and in combination with Herceptin (trastuzumab )[95]. In this study, neither GSI had any effect on tumor volume as single agents or in combination with Herceptin. Herceptin alone had dramatic effects, leading to apparently complete tumor regression. However, when tumor recurrence was studied, MRK003 in combination with Herceptin completely abolished tumor regression, while LY411575 nearly abolished it. Animals treated with Herceptin alone showed approximately 50% tumor recurrence after complete regression, which is similar to patients treated with Herceptin-containing regimens. Thus, it is likely that the effects of GSIs and potentially other Notch inhibitors may be clinically very significant in terms of long-term survival, but will have to be evaluated with appropriate surrogate endpoints and in mechanism-based, rationally designed combinations. In fact, trials designed to combine GSIs with other agents, including tyrosine kinase inhibitors, mammalian target of rapamycin inhibitors, aromatase inhibitors, and conventional chemotherapeutic compounds are currently recruiting patients [96]. Consistent with this view, in a recently closed US National Cancer Institute sponsored phase I/II study at the Memorial Sloan-Kettering Cancer Center, (New York, NY, USA) (NCT01154452), RO4929097 has been administered in combination with a hedgehog inhibitor, GDC-0449, for the treatment of adult patients with advanced and/or metastatic sarcomas, including SS. The objectives of this study were to determine the maximum-tolerated dose of RO4929097 combined with GDC-0449 (Phase Ib) and to assess the progression-free survival of patients treated with RO4929097 alone or with the hedgehog antagonist (Phase II). Studies like this may hold the key to future development of Notch-modulating agents in pediatric sarcomas, and for that matter in other tumors.

**Table 2:** Completed clinical trials with γ-secretase inhibitors in pediatric/young adult oncologic patients (clinicaltrials.gov).

Compound	ClinicalTrials Gov Identifier	Clinical studies	Cancer type	Patients'age
MK0752	NCT00106145	Phase I study	Breast and advanced solid tumors	18 Years and older
MK0752	NCT00100152	Phase I study	T-ALL	12 Months and older^a^
RO4929097	NCT01192763	Phase I study	Pancreatic cancer	18 Years and older
RO4929097	NCT01208441	Phase I study	Breast cancer	18 Years and older
RO4929097	NCT01269411	Phase I study	Brain and Central Nervous System Tumors	18 Years and older
RO4929097	NCT01216787	Phase II study	Melanoma (Skin)	18 Years and older
RO4929097	NCT01217411	Phase I study	Breast Cancer	18 Years and older
RO4929097	NCT01151449	Phase II study	Breast Cancer	18 Years and older
RO4929097 with or without Bevacizumab	NCT01270438	Phase II study	Metastatic Colorectal Cancer	18 Years and older
RO4929097	NCT01236586	Phase I/II study	Brain and Central Nervous System Tumors, T-ALL	1 Year to 21 Years^a^
RO4929097	NCT01088763	Phase I/II study	Leukemia	1 Year to 21 Years^a^

**^a^**Enrollment of children. T-ALL, T-cell acute lymphoblastic leukemia/lymphoma.

## Conclusions

Taken together, the results we have summarized suggest that Notch inhibition may be a promising approach in the treatment of several types of human pediatric cancers, but more work needs to be done to assure a successful clinical translation. The role of individual Notch paralogs in specific tumor subtypes will dictate whether a relatively non-specific approach such as a GSI or more specific agents, such as Notch receptor or ligand monoclonal antibodies, should be preferred [44]. Not surprisingly, several academic and pharmaceutical groups are developing specific Notch receptor and Notch ligand antibodies [77]. In tumors in which Notch signaling plays a tumor-suppressive role, such as ES [51,52,54], agents that can selectively activate Notch receptors or induce downstream molecules such as HEY1 may be promising therapeutic approaches.

Overall, preclinical studies suggest that an anti-cancer approach aimed at promoting differentiation in STS is feasible and deserves further investigation. Given the prominent role of Notch signaling in numerous aspects of tumor development and maintenance, the Notch pathway is a potentially attractive therapeutic target for several types of STS.

## Abbreviations

ATF2: activating transcription factor 2; CDK: cyclin-dependent kinase; CNS: central nervous system; CSL: DNA binding protein transcriptional complex; DLL1: Delta-like 1; DLL3: Delta-like 3; DLL4: Delta-like 4; EGR1: Early Growth Response-1; ES: Ewing sarcoma; ETS: E transformed specific transcription factor; EWS: Ewing sarcoma breakpoint region; EZH2: Enhancer of Zeste Homolog 2; FLI1: Friend leukemia virus integration 1; FOXO1: forkhead box O1; GSI: γ-secretase inhibitor; HDAC: histone deacethylase; ICD: Notch intracellular domain; LFNG: Lunatic Fringe enzyme; LSD1: lysine-specific demethylase 1; MFNG: Manic Fringe enzyme; MSC: mesenchymal progenitor/stem cell; PAX3: paired box 3; PAX7: paired box 7; PEST: C-terminal proline: glutamic acid: serine and threonine degradation domain; PRC2: Polycomb Repressor Complex 2; PTEN: Phosphatase and Tensin homolog deleted in chromosome 10; QRT-PCR: quantitative reverse transcriptase-polymerase chain reaction; RBP-Jk: Recombination signal Binding Protein for immunoglobulin Kappa J region; RFNG: Radical Fringe enzyme; RMS: rhabdomyosarcoma; SIRT1: sirtuin 1; SS: synovial sarcoma; SS18: synovial sarcoma translocation: chromosome 18; SSX1: Synovial Sarcoma: × breakpoint 1; SSX2: Synovial Sarcoma: × breakpoint 2; SSX4: Synovial Sarcoma: × breakpoint 4; STS: soft tissue sarcoma; T-ALL: acute T-cell leukemia; TGFβ: tumor growth factor β; TLE: Transducin-like Enhancer of split genes.

## Competing interests

The authors declare that they have no competing interests.

## Authors' contributions

RR conceived the outline of the manuscript, selected the literature, and wrote the manuscript. RC contributed to the selection of literature on clinical trials and to the initial version of the manuscript. FL and LM contributed to the discussion on clinical implications and critically reviewed the manuscript. All authors read and approved the final version of the manuscript.

## Authors' information

RR is a PhD and the Group leader of the Laboratory of Angiogenesis with experience in mechanisms that regulate gene expression and cell growth in pediatric cancers. RC is a PhD working on transcriptional regulation in cancer in the Laboratory of Angiogenesis directed by RR. LM is an MD and the Director of the Cancer Centre and Professor of Medicine and Pharmacology at the Jackson University which is committed to preclinical and clinical research against breast cancer. FL is an MD and Full Professor of Pediatrics and the Head of the Oncohematology Department with a long-standing experience in preclinical research and clinical management of pediatric tumor patients.

## Pre-publication history

The pre-publication history for this paper can be accessed here:

http://www.biomedcentral.com/1741-7015/10/141/prepub
